# Evolution of Surgical Flap Delay and Conditioning in Reconstructive Surgery

**DOI:** 10.7759/cureus.116456

**Published:** 2026-09-17

**Authors:** Merlin Basheva

**Affiliations:** 1 Department of Thermal Trauma, Plastic, Reconstructive and Aesthetic Surgery, Military Medical Academy, Varna, BGR

**Keywords:** delay phenomenon, facial reconstruction, flap, flap conditioning, paramedian forehead flap, plastic surgery, reconstructive surgery, scalp reconstruction, surgical flap delay

## Abstract

Surgical delay is a staged technique in which selected vascular connections are interrupted before definitive flap transfer, allowing time for vascular adaptation. This narrative historical review examines its development, experimental physiology, and current use with perfusion assessment, using craniofacial and scalp reconstruction as the principal clinical example. A targeted search of PubMed/MEDLINE (Medical Literature Analysis and Retrieval System Online), publisher archives, and the reference lists of landmark reviews was performed up to August 7, 2026, with emphasis on primary historical reports, mechanistic studies, craniofacial applications, and recent clinical syntheses. The historical record shows three overlapping phases. Early staged reconstructions incorporated deliberate delay before its physiology was understood. Delay then became a reproducible experimental phenomenon, initially explained by denervation, adrenergic recovery, and metabolic adaptation; classic animal studies later demonstrated early increases in flow through dilating and remodeling pre-existing choke vessels. Molecular signaling and capillary neovascularization have been observed in selected tissues and time frames, but their causal contributions remain uncertain. In contemporary practice, conventional surgical delay remains selective rather than routine. Indocyanine green angiography may supplement clinical evaluation and fixed intervals before paramedian forehead flap division; published near-infrared spectroscopy experience in forehead flaps remains limited to two cases. Most evidence consists of animal studies, retrospective series, or small feasibility cohorts, and remote ischemic, pharmacologic, cellular, and exosome-based conditioning remain unproven for routine clinical use. Current evidence does not establish a universal interval, a validated perfusion threshold, or a routine clinical role for newer conditioning methods.

## Introduction and background

A flap is tissue transferred to a defect while its blood supply is maintained or surgically re-established. In a pedicled flap, that supply enters through a pedicle that remains connected to the donor site; the recipient bed is the wound surface into which the flap is set. Each flap has a dependable perfusion territory, and tissue carried beyond it, or transferred into a poorly vascularized bed, may become ischemic.

Surgical delay seeks to improve vascular reliability before definitive transfer, at the cost of an additional operation and a longer treatment course. During surgical delay, selected vascular inputs to tissue intended for later transfer are interrupted at a preliminary operation. The interval before transfer permits adaptation to a subsequent ischemic challenge. The technique predates vascular imaging and microsurgery but remains relevant in selected staged facial reconstruction and newer tissue-conditioning strategies [1,2].

Several related procedures now share the language of conditioning. Classical surgical delay alters a flap’s vascular connections days or weeks before transfer. Ischemic preconditioning usually means brief cycles of ischemia and reperfusion shortly before a major insult. Remote ischemic conditioning applies those cycles to another body region. Temporary pedicle occlusion before division tests a transferred flap’s tolerance of reduced pedicle inflow; division is the definitive detachment. These maneuvers may recruit overlapping protective pathways, but they are not synonyms and should not be judged by the same evidence [2].

This review aims to show how an empirical surgical habit became a physiological concept, and to ask what the history means for current reconstructive decisions. Facial and scalp reconstruction are emphasized because staged tissue transfer remains clinically important in these regions, including historical attached arm flaps and current fluorescence-guided forehead-flap division. The broader literature is included when it clarifies mechanism or reveals the limits of translation. The primary question is whether pre-transfer delay and its contemporary surrogates change the reliability of staged tissue transfer and, if so, how that change is now judged. Craniofacial and scalp reconstruction serve as the principal clinical test case; the review is not an exhaustive comparison of conditioning interventions across all reconstructive sites, and free-flap perfusion monitoring and ischemia-reperfusion pharmacology without a flap endpoint are outside its scope.

Earlier reviews compiled delay knowledge across specialties or surveyed preconditioning mechanisms, but they largely predate the recent craniofacial perfusion studies, minimally invasive approaches, and clinical trials considered here. This review distinguishes pre-transfer delay from assessment of post-transfer recipient-site perfusion and from other conditioning methods, treats disputed priority claims cautiously, and evaluates whether contemporary perfusion assessment has altered clinical decision-making.

## Review

Search strategy and historical method

A targeted narrative search of PubMed/MEDLINE (Medical Literature Analysis and Retrieval System Online) from database inception, supplemented by publisher archives and backward and forward citation searching, was last updated on August 7, 2026. Search concepts included “delay phenomenon,” “surgical delay,” “vascular delay,” “flap training,” “flap conditioning,” “ischemic preconditioning,” “forehead flap division,” “expanded forehead flap,” “nasolabial flap,” “prelamination,” “scalp flap,” “indocyanine green,” “near-infrared spectroscopy,” “botulinum toxin,” “extracellular vesicles,” and “exosomes.” Primary experimental and clinical reports were favored for milestone claims; recent systematic or scoping reviews were favored for contemporary evidence. Historical works were retained when they documented an operation, terminology, or experimental turning point; conditioning studies without a flap-related endpoint were included only when they clarified development of the concept. The review was informed by the six Scale for the Assessment of Narrative Review Articles (SANRA) domains: justification, aims, description of the literature search, referencing, scientific reasoning, and appropriate presentation of data [3]. Search terms were in English, and screening and selection were performed without duplicate assessment. This was not a systematic review, and no meta-analysis or formal risk-of-bias assessment was performed.

Definitions of delay and conditioning

At its narrowest, delay is a planned preliminary operation that reduces selected vascular inputs while the tissue remains attached to a dependable source. The later flap can therefore include a larger effective territory or tolerate transfer more reliably. At its broadest, conditioning includes any controlled stimulus intended to improve perfusion, ischemic tolerance, or resistance to ischemia-reperfusion injury. The broad definition is useful for mapping a field, but dangerous if it makes an old operative maneuver appear equivalent to a laboratory injection or a blood-pressure cuff protocol. Table 1 separates the terms by what is actually done and what is being inferred.

**Table 1:** Related terms that should not be used interchangeably

Term	What is done	Intended endpoint	Interpretive caution
Surgical delay	The flap is partly incised, elevated or undermined and/or selected vessels are divided before definitive transfer.	Enlarge the dependable perfused territory and improve later survival.	Classical operation performed over days to weeks; technique and interval are flap-specific [1,2].
Arterial vascular delay	A named arterial inflow is ligated or embolized before flap harvest.	Redirect arterial inflow and stimulate intraflap vascular adaptation.	Venous delay alters outflow and should be reported separately; technique and intended vascular target must be explicit [1,2].
Staged pedicle division	A transferred pedicled flap is partially or fully detached after an interval for recipient-site vascularization.	Complete transfer after adequate recipient-site perfusion has developed.	Related to delay biology, but occurs after inset and does not directly demonstrate complete vascular independence [1].
Flap training	The pedicle is intermittently clamped or narrowed before division.	Test tolerance of reduced pedicle flow and, in some protocols, promote recipient-site perfusion.	A descriptive clinical term; protocols and endpoints vary.
Ischemic preconditioning	Brief local ischemia-reperfusion cycles precede a longer ischemic insult.	Increase tolerance to subsequent ischemia-reperfusion injury.	Usually minutes to hours, not the same operation or time scale as surgical delay [2].
Remote ischemic conditioning	Brief ischemia-reperfusion cycles are applied to a distant limb.	Trigger systemic protective signaling.	Operationally distinct from surgical delay; clinical efficacy requires separate testing [2].
Pharmacologic, biologic, or physical conditioning	Drugs, growth factors, cells, heat, or other stimuli are applied.	Mimic vascular or cytoprotective effects without an operation.	A diverse family rather than a single established therapy [2].

How surgical delay is performed

Delay is not one standardized operation. In a classical random-pattern flap, the surgeon may incise part of the perimeter, elevate part or all of the planned flap, or divide selected vascular connections while preserving a dependable base. When the flap is subsequently transferred, it is expected to perfuse a territory that would have been unreliable at the first operation. The extent of preliminary elevation matters: partial or complete elevation interrupts more vascular connections than marginal incision alone. Because effective technique varies by flap design and experimental model, the preserved attachments and the extent and direction of elevation should be reported explicitly [1,2].

In an axial or perforator-based flap, delay may instead remove competing inflow, interrupt selected perforators, ligate a named vessel, or redirect flow toward the vascular axis intended to support the definitive reconstruction. Venous delay deliberately alters outflow and should be reported separately because venous congestion, arterial redistribution, and ischemia-reperfusion injury are not interchangeable physiological problems. Tissue expansion adds another layer: expansion may enlarge and reorganize the vascular network, but it also permits longer and sometimes narrower-based designs, for which an additional delay may still be chosen [1,2].

Finally, staged pedicle clamping or division after a flap has been inset assesses whether recipient-site perfusion is sufficient rather than performing a conventional pre-transfer delay. Its biological relationship to delay is real, but its clinical endpoint is different. This distinction is particularly important in facial reconstruction, where “delay,” “training,” and “early division” are sometimes used loosely for different stages of the same reconstruction. Technique, timing, and intended endpoint should therefore be stated explicitly whenever outcomes are compared.

Historical development and experimental study

Early reconstructive procedures are best regarded as antecedents of surgical delay rather than descriptions of its mechanism. Nasal reconstruction was practiced before the Renaissance, including Indian reconstructive traditions and the Italian arm-flap method; the precise transition from cheek-based to forehead-based Indian techniques is not securely documented. In 1597, Gaspare Tagliacozzi published the first comprehensive book devoted to reconstructive plastic surgery and described an arm flap that remained attached until a later stage [4,5]. The account describes deliberate staged preparation of the flap before transfer, although Tagliacozzi did not formulate a physiological explanation for the delay response. First performance, first publication, first naming, and first mechanistic experiment are therefore different historical claims.

A further account of intentional pre-transfer delay appeared in 1854, when Frank Hastings Hamilton described elevating a broadly attached calf flap, waiting about two weeks before transfer, and dividing the pedicle later [6]. It is defensible to call this an early, well-documented surgical delay, but the surviving record does not establish it as the first such operation. Hamilton’s report is important because it gives an unusually detailed clinical account of the waiting period and its intended purpose.

Staging became a disciplined reconstructive strategy during and after the First World War. Filatov, Ganzer, and Gillies appear to have developed tubed-pedicle methods independently, protecting the raw surface of long flaps and permitting tissue to travel across several operations [7]. Blair’s 1921 paper explicitly described delayed transfer of long pedicle flaps, bringing the maneuver into a recognizable modern surgical literature [8]. Staged transfer allowed long flaps to remain perfused while they acquired circulation at successive recipient sites.

Objective assessment began before the classic animal models. In 1943, Douglas and Buchholz described a method for testing circulation in pedicle flaps, illustrating the broader transition from color, temperature, and bleeding at an edge toward measured vascular sufficiency [9]. Such tests did not yet explain delay, but they changed the type of question that could be asked: not simply whether a flap looked viable, but how efficiently its pedicle carried blood and when division might be tolerated.

Reproducible experimental investigation began in the 1960s. Two studies by McFarlane et al., published in 1965, are often conflated: one tested the delay phenomenon in experimental pedicle flaps [10]; the other introduced the standardized dorsal rat flap used to study necrosis and its prevention [11]. These papers should be distinguished because one examined the conditioning maneuver, whereas the other established a model used in subsequent flap-survival research.

Myers and Cherry subsequently demonstrated that preliminary delay augmented survival in rabbit flaps and investigated its early mechanism [12,13]. Milton then compared several delay methods and intervals in pigs, finding flap elevation more effective than marginal incision alone and identifying approximately two weeks as the most effective interval in that particular model [14]. These studies showed that both method and interval influenced survival and that timing derived from one model should not be applied universally. Regional blood flow, vessel caliber, metabolism, histology, and timing could now be studied separately. Selected clinical and experimental milestones are summarized in Figure 1.

**Figure 1:**
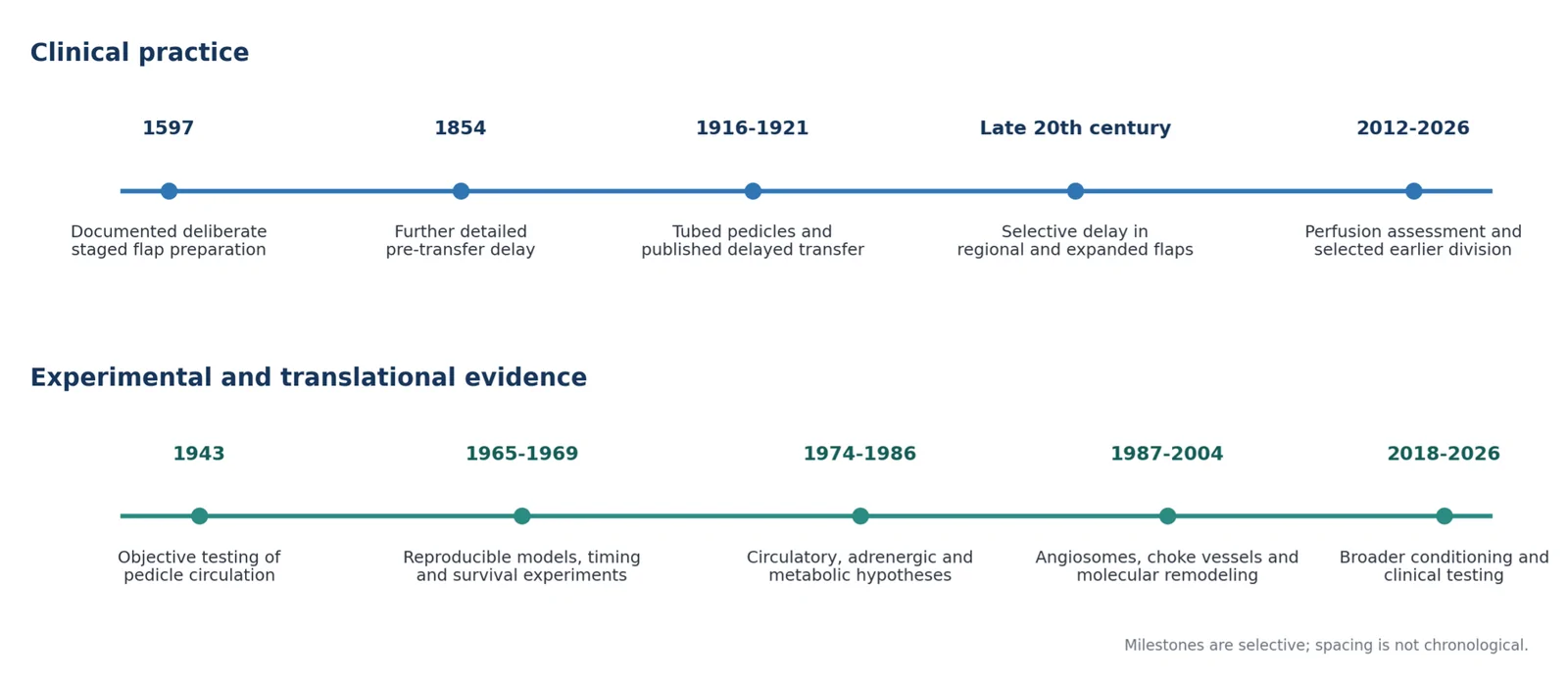
Clinical and experimental milestones in surgical flap delay Clinical staging preceded mechanistic study by centuries. Historical and early experimental milestones are based on sources [1,2,4-14]; later developments are discussed and referenced in the corresponding sections. Figure created by author using Python (matplotlib; Python Software Foundation, Wilmington, Delaware, United States)

Proposed biological mechanisms

Early explanations focused on the circulatory disturbance produced by flap elevation. The blood vessels of skin tubes and flaps were under investigation from the earliest years of the specialty [15]. Reinisch subsequently proposed that sympathetic denervation opened non-nutritive arteriovenous shunts after flap elevation and that delay permitted their subsequent closure [16]. Pearl described recovery from a transient hyperadrenergic state after norepinephrine release [17]. Subsequent work did not establish arteriovenous shunting or adrenergic recovery as a complete explanation. Metabolic studies also showed adaptation: in delayed experimental flaps, glucose utilization and hexokinase activity changed during the interval before transfer [18]. These findings support metabolic involvement but do not establish a single dominant mechanism.

Anatomical and serial-flow studies provided stronger evidence, principally in classic animal models. In pigs, blood flow rose by the second day and increased sharply between days two and three without a parallel increase in arterial density over the same short interval [19]. The angiosome concept then supplied a three-dimensional map: adjacent source-artery territories may be connected either by reduced-caliber choke anastomoses or by similar-caliber true anastomoses [20]. Classic experimental delay studies, supported by clinical anatomic observations, showed preferential dilation and remodeling of the choke vessels, lowering resistance across a territorial boundary and allowing the flap to recruit adjacent territory [21,22]. In this review, “linking vessels” refers principally to these choke connections.

Timing studies strengthened that account without establishing a universal timetable. In a rabbit model, the fastest increase in choke-vessel caliber occurred approximately 48-72 hours after delay [23]. Larger rabbit and canine experiments found that an initial vasoconstriction resolved within hours, followed by dilation, wall remodeling, and persistent structural change [24]. These experimental intervals should not be extrapolated directly to human pedicle division. Flap design, tissue type, expansion, comorbidity, recipient bed, and the exact delay maneuver all alter the clinical problem.

Later molecular work showed that the response is not limited to pre-existing vessels. In a rat transverse rectus abdominis myocutaneous flap model, delay increased flap survival and expression of vascular endothelial growth factor and basic fibroblast growth factor messenger RNA; histological sections also showed distal neovascularization [25]. Taken together, classic experimental studies indicate that early increases in flow can precede detectable increases in arterial density and coincide with dilation and remodeling of pre-existing choke vessels. Metabolic changes, growth-factor expression, and distal neovascularization have also been observed in selected models, but their relative causal contributions vary and have not been established across flap types [18-25]. Modern taxonomies therefore place surgical delay within a broader and less clinically mature family of tissue-conditioning strategies [26]. The proposed mechanisms are summarized in Figure 2.

**Figure 2:**
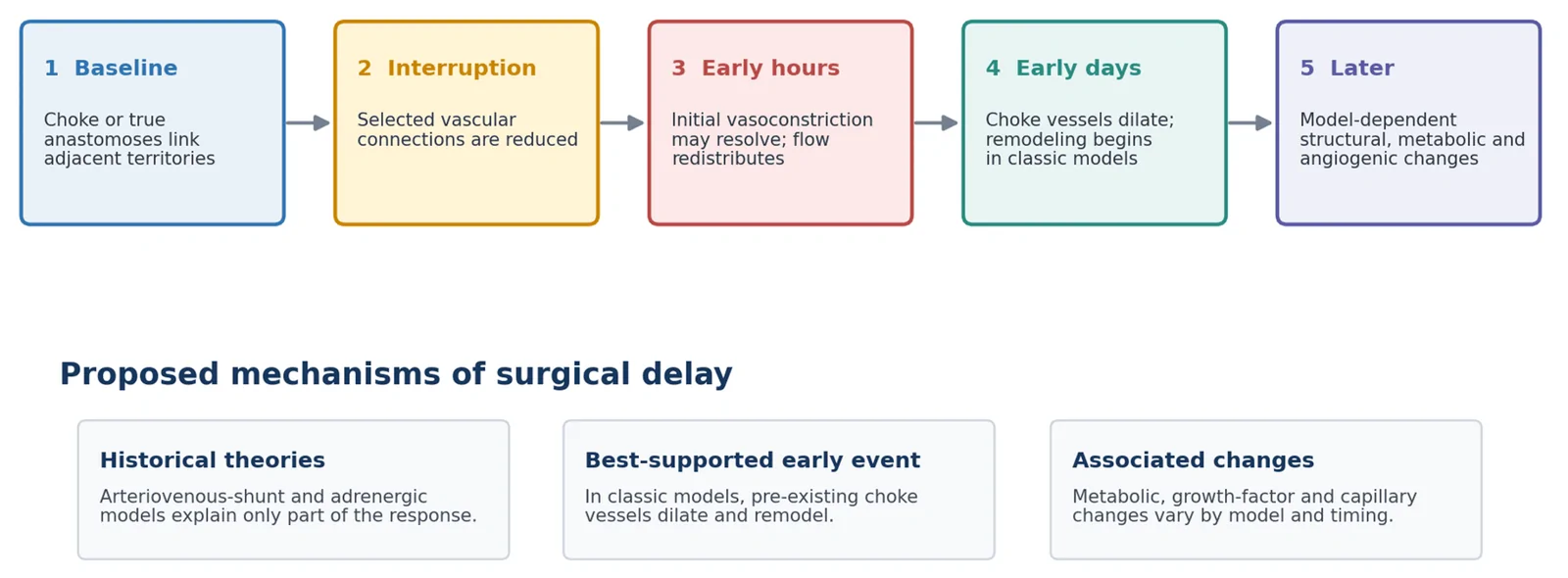
Proposed mechanisms of classic surgical delay Conceptual flow chart of classic incisional and arterial delay; venous delay has distinct congestion biology. Timings and associated changes are based largely on classic experimental models and do not define a clinical timing algorithm. Timing and dominant pathways vary with flap design, species, tissue expansion and the conditioning maneuver, and experimental intervals are not universal clinical cutoffs. Boxes 1-4 summarize findings from classic animal models, with human support limited to angiographic and perfusion-imaging observations; the changes in box 5 are model-dependent and partly inferential [15-25]. Figure created by author using Python (matplotlib; Python Software Foundation, Wilmington, Delaware, United States)

Craniofacial and scalp applications

Staged facial reconstruction provides a direct clinical example because the flap remains visible between operations. The paramedian forehead flap is transferred to the nose on a vascular pedicle and later divided after recipient-site perfusion has developed. This is not identical to pre-transfer surgical delay. The practical question is whether recipient-site perfusion is sufficient for pedicle division. Traditionally, timing has been based on a customary interval modified by clinical examination.

Indocyanine green (ICG) angiography permits assessment of relative flap perfusion after temporary pedicle occlusion. An early prospective study of six patients found ICG evidence consistent with recipient-bed vascular ingrowth as early as one week after transfer [27]. A subsequent 10-patient series used ICG to support division at two weeks in selected partial-thickness defects with a substantially vascularized recipient bed and no nicotine use [28]. In a later cohort of 71 forehead flaps, quantitative ICG measurements after temporary pedicle clamping showed relationships between interstage time, cartilage grafting, and perfusion behavior [29]. A 10-patient feasibility series reported ICG-assisted division at seven days in strictly selected partial-thickness defects [30]. Another 10-patient prospective series assessed perfusion between days 14 and 25; only two flaps were divided earlier, on days 14 and 16, and one developed a minor wound-healing problem [31]. Dye-free near-infrared spectroscopy has also been used to map tissue oxygen saturation in forehead flaps, although published evidence is limited to two cases and no validated threshold exists [32].

A 2025 systematic review and institutional series assembled 103 early forehead-flap divisions. No complete flap loss was reported, and two cases of epidermal necrosis healed conservatively; in a series of this size, absence of observed flap loss does not establish a negligible risk. Patients were selected, many defects were partial thickness, and imaging was not used uniformly [33]. A separate 2026 retrospective cohort included 141 interpolation flaps, of which 28 were paramedian forehead flaps and 14 of those were divided before 21 days. Complication rates did not differ significantly between early and conventional takedown in the overall cohort, a finding that does not establish equivalence, particularly for the 14 early forehead flaps; timing was not imaging-guided, and the design remains vulnerable to selection bias [34]. A 2026 meta-analysis of seven retrospective cohorts (428 patients) found no significant differences between two- and three-stage forehead flaps in infection, wound breakdown, necrosis, or revision, with low certainty of evidence; none of the pooled studies recorded whether the intermediate stage was chosen for vascular conditioning or for staged thinning, so the contribution of the delay component cannot be isolated [35]. These findings support consideration of earlier division in selected cases. Perfusion assessment may supplement clinical examination, but neither a universal interval nor a validated physiological threshold has been established.

Earlier division may also reduce patient burden. The visible pedicle can be markedly disfiguring and can interfere with daily activities; in one 26-patient series, 94% preferred division at approximately one week rather than three [36]. Patient-reported outcomes, treatment inconvenience, and cost were infrequently reported in the studies reviewed. An imaging strategy should demonstrate that it can shorten treatment or reduce uncertainty without increasing partial loss, unplanned revision, or anxiety. This patient-centered endpoint is especially relevant for facial reconstruction, where the temporary stage is publicly visible.

Two 2026 retrospective facial series illustrate continued use of planned delay in complex nasal reconstruction. Qiang et al. described 30 expanded forehead flaps combined with local turnover scar flaps in a staged protocol that included flap delay three weeks after transfer and pedicle division one week later; no major complication was reported, but the series had no undelayed comparator [37]. Demirbaşoğlu et al. reported 22 delayed and prelaminated nasolabial flaps; all survived without total loss, although two developed partial distal necrosis, two developed venous congestion, and six required revision procedures [38]. These reports support feasibility but do not establish the independent contribution of delay.

The scalp contributes a different problem: hair-bearing tissue is scarce, relatively inelastic, and sometimes expanded before transfer. In a 43-patient series of pre-expanded scalp flaps for facial defects, post-transfer pedicle delay and division were used in the subgroup receiving pedicled flaps, but the retrospective design could not isolate their effect [39]. In a pediatric series of 66 tissue-expansion procedures, delay was used in 17 less favorably designed flaps; only five delayed procedures involved the head and neck [40]. These reports support selective feasibility in difficult geometry, not superiority over an undelayed flap.

Direct evidence for delayed reconstruction of scalp defects is limited but relevant. A four-patient series used three-stage elevation of bipedicled scalp flaps at four-week intervals before custom cranioplasty; no infection, implant exposure, or flap necrosis occurred during six to 12 months of follow-up, although one patient developed flap congestion and wound dehiscence after the scalp was expanded by the skull reconstruction [41]. A 2024 retrospective series used pre-transfer delay to extend lower trapezius myocutaneous flaps for seven full-thickness posterior scalp defects, all of which healed without a reported complication [42]. Neither series had an undelayed comparator. A 2026 randomized study of 40 patients with large upper facial or scalp lesions found that adding botulinum toxin to fronto-scalp expansion and laser therapy improved expansion efficiency and reduced postoperative retraction, without significant differences in pain or complications; it did not test flap survival or establish a vascular delay effect [43].

Interpretation and limits of perfusion assessment

These studies tested whether perfusion assessment can supplement fixed intervals. Temporary pedicle clamping with ICG angiography or near-infrared spectroscopy assesses relative perfusion or tissue oxygenation; it does not directly demonstrate recipient-bed vascular ingrowth or complete vascular independence, and validated decision thresholds have not been established [27-32]. Imaging does not itself condition the tissue.

The same technology has been applied to the delay maneuver itself rather than to division readiness: in two high-risk head and neck patients, quantitative ICG imaging at flap elevation and again after a three-week vascular delay documented increased perfusion, a two-patient pilot rather than comparative evidence [44]. Outside the face, office-based ICG angiography with the pedicle under tourniquet has been used to time division of delayed reverse sural flaps in two limb-salvage patients, postponing division in one when distal opacification was inadequate and confirming readiness in the other [45].

Evidence remains too limited for a universal numerical cutoff. Current forehead-flap studies are small, often single-center, and enriched for favorable anatomy. Measurements depend on acquisition and analysis methods, and published studies have used different qualitative and quantitative approaches. Future trials should predefine the clinical decision, imaging protocol, threshold, rescue rule, and patient-centered outcomes.

Minimally invasive and experimental conditioning strategies

Microsurgery reduced the need to delay many conventional flaps, but it also widened interest in less invasive conditioning. Abdominal-flap arterial vascular delay can be performed surgically or by preoperative endovascular embolization. A 2024 scoping review found only nine clinical studies, substantial protocol heterogeneity, and no agreed optimal method, although selected high-risk patients may benefit [46]. A 2025 systematic review of minimally invasive perfusion optimization found six human studies across angiographic delay, heat, and ischemic approaches; it included only one ischemic-preconditioning study, which found no significant benefit [47].

Remote ischemic conditioning is attractive because a blood-pressure cuff on a limb could, in theory, trigger systemic protection without touching the flap. Clinical head and neck evidence remains limited and largely neutral. In a randomized trial of 60 patients undergoing microsurgical reconstruction, 30-day flap failure occurred in two of 30 conditioned and one of 30 sham-treated patients (relative risk 2.0, 95% CI 0.2-20.9), an estimate too imprecise to exclude clinically important benefit or harm; pedicle thrombosis was likewise not demonstrably reduced [48]. Another randomized trial found similar flap tissue oxygen saturation on the first postoperative day with conditioning and sham (85 ± 12% versus 83 ± 9%; P = 0.471) [49]. In a small 2025 trial restricted to previously irradiated patients, flap oxygenation again showed no demonstrable improvement; a lower rate of composite complications was a secondary finding and should be treated as hypothesis-generating rather than confirmatory [50]. Collectively, these trials have not demonstrated reproducible clinical benefit, and their non-significant results should not be read as evidence of equivalence.

Other investigated methods include pharmacologic vasodilation, growth factors, stem-cell products, extracellular vesicles, thermal stimuli, and shock waves [26,51]. Most evidence remains preclinical, and standardized human protocols are lacking. Botulinum neurotoxin has sometimes been described as a form of “chemical delay,” although its effects should not be assumed to reproduce surgical vascular remodeling. A systematic review identified 36 controlled animal studies involving 1,032 animals; 29 studies contributed quantifiable data to the meta-analysis, which found increased perfusion, vessel caliber, and survival of random-pattern and pedicled flaps [52]. Protocols were heterogeneous, blinding and animal-death reporting were inconsistent, and no consistent optimal pretreatment interval emerged. The 2026 fronto-scalp trial evaluated expansion and retraction rather than flap survival [43]; an expansion-related benefit was reported in that protocol, but botulinum toxin remains investigational as a vascular-conditioning strategy.

A 2026 systematic review and meta-analysis of exosomes included 24 animal studies and reported apparent improvements in flap or graft survival, alongside extreme heterogeneity and indicators of small-study and publication effects [53]. Exosome therapy therefore remains preclinical and is not ready for routine clinical use. Table 2 compares the evidentiary maturity and principal limitations of the contemporary approaches.

**Table 2:** Evidence and limitations of contemporary conditioning approaches

Approach	Representative evidence	What can reasonably be said now	Main limitation	
Conventional surgical delay	Historical clinical experience, anatomical work, and animal mechanistic studies [1,21,22,24].	A valid selective option when flap geometry or vascular risk is unfavorable.	No universal technique or interval; much mechanistic evidence is species- and model-dependent.	
Expanded and delayed craniofacial/scalp flaps	Retrospective and small clinical series plus one fronto-scalp expansion trial [37-43].	Staged approaches are feasible in selected complex defects; one trial reported improved expansion efficiency with botulinum toxin.	Indirect, uncontrolled evidence from few patients and heterogeneous staged procedures; no comparative proof that delay itself improves survival, and the expansion trial did not test flap survival or isolate a vascular-delay effect.	
Perfusion-guided pedicle division	Indocyanine green (ICG) cohorts and small prospective/case series; 2025 synthesis of 103 early divisions [27-33].	Can document relative perfusion and has accompanied earlier division in selected series; validated thresholds and evidence of improved outcomes remain unavailable.	No standardized acquisition method or validated safety threshold.	
Abdominal vascular or angiographic delay	Nine-study scoping review; minimally invasive systematic review [46,47].	Feasible and potentially useful in selected patients; comparative benefit and the optimal technique remain uncertain.	Heterogeneous procedures, populations, and endpoints.	
Remote ischemic conditioning	Small randomized head and neck trials [48-50].	Primary oxygenation and hard-outcome results remain neutral; one irradiated-cohort trial found a favorable secondary composite signal.	Underpowered trials and variable protocols; clinical efficacy remains unproven.	
Pharmacologic/cellular conditioning	Broad preclinical review and botulinum-toxin animal meta-analysis [26,51,52].	Pooled animal findings suggest vascular effects of botulinum toxin; a clinical vascular-conditioning role has not been established.	No established human vascular-conditioning dose, timing, or flap-survival efficacy.	
Exosome therapy	2026 meta-analysis of 24 preclinical animal studies [53].	Preclinical studies report improved survival, but replication and standardization are required.	High heterogeneity, reporting weaknesses, and no clinical efficacy data.	

Practical implications for surgical planning

Clinical use should remain selective. Pre-transfer delay may be considered when a planned flap is unusually long or narrow, crosses a vascular boundary, uses expanded tissue, or has vascular inputs altered by previous surgery; these circumstances are rational considerations rather than validated indications [1,22,24,38,40-42]. Within the evidence reviewed here, nicotine exposure, diabetes, and prior radiotherapy were not established as stand-alone indications for delay. Because delay adds another operation, the expected vascular benefit should be explicit and a simpler or more dependable reconstruction should still be preferred when available.

Reports should document flap dimensions and tissue layers, the vascular inputs preserved and interrupted, the extent of preliminary elevation, the interval, recipient-bed characteristics and patient risk factors, and any clinical or objective perfusion assessment used before transfer or pedicle division. For perfusion-guided division, the temporary-occlusion method, imaging acquisition, reference region, decision rule, and rescue plan should also be specified. These details are more transferable than a single calendar interval.

What is established and what remains uncertain

Three conclusions are supported by current evidence. First, partial preliminary interruption can increase the territory that some flaps reliably perfuse. Second, in classic animal models, early increases in flow through pre-existing vascular connections precede or accompany later remodeling; model-dependent metabolic, molecular, and angiogenic changes have also been observed. Third, no universal interval applies across flap types. A result from a rabbit skin flap, a delayed abdominal perforator flap, and a forehead flap acquiring recipient-bed perfusion cannot be converted into one timetable.

Four evidentiary levels should remain separate. Experimental flap-survival area demonstrates biological potential, but may depend on species, flap dimensions, and the method used to define necrosis. A physiological surrogate such as fluorescence ingress, tissue oxygen saturation, or microvascular flow can document change without proving fewer clinical complications. A clinical series can show feasibility but is vulnerable to selection of favorable patients and experienced operators. Only comparative clinical studies can determine whether conditioning or image-guided timing improves outcomes that matter to patients. Much apparent disagreement in this literature results from asking different questions with these different endpoints rather than from truly contradictory biology.

Future clinical studies should define the maneuver precisely, separate pre-transfer delay from post-transfer pedicle testing, stratify by smoking, diabetes, prior radiotherapy, tissue expansion, cartilage grafting, and defect thickness, and use reproducible perfusion acquisition. Outcomes should include partial necrosis, unplanned reoperation, total treatment time, patient burden, cost, and the consequences of a falsely reassuring test. Negative results should also be reported.

This review also has limitations. Its narrative selection was purposive rather than exhaustive, screening was not duplicated, and no formal risk-of-bias assessment was performed. The search used English-language terms, and reliance on PubMed/MEDLINE and accessible publisher archives may underrepresent non-indexed or non-English historical material. The clinical conclusions are therefore qualitative and may be affected by publication and selection bias.

Historical priority claims should distinguish first performance, publication, terminology, and mechanistic investigation without assuming that early staged procedures anticipated modern conditioning methods.

## Conclusions

Surgical flap delay evolved from deliberately staged reconstruction performed before its physiology was understood into an experimental model of vascular adaptation and a selective clinical technique. Classic experimental studies support early redistribution of flow through pre-existing vascular connections, followed by model-dependent structural, metabolic, molecular, and angiogenic changes.

Recent clinical work has focused on perfusion assessment before pedicle division. In staged facial reconstruction, ICG angiography can assess relative perfusion and near-infrared spectroscopy has been reported in very small series, but no threshold has been validated as universal and outcome benefit remains uncertain. Direct evidence for pre-transfer delay in scalp reconstruction is limited to small uncontrolled series. Conventional delay therefore remains selective, while remote ischemic, pharmacologic, cellular, and exosome-based conditioning remain investigational.
